# Artificially Edited Alleles of the Eukaryotic Translation Initiation Factor 4E1 Gene Differentially Reduce Susceptibility to Cucumber Mosaic Virus and Potato Virus Y in Tomato

**DOI:** 10.3389/fmicb.2020.564310

**Published:** 2020-12-10

**Authors:** Hiroki Atarashi, Wikum Harshana Jayasinghe, Joon Kwon, Hangil Kim, Yosuke Taninaka, Manabu Igarashi, Kotaro Ito, Tetsuya Yamada, Chikara Masuta, Kenji S. Nakahara

**Affiliations:** ^1^Research and Development Division, Kikkoman Corporation, Noda, Chiba, Japan; ^2^Research Faculty of Agriculture, Hokkaido University, Sapporo, Japan; ^3^Department of Agricultural Biology, Faculty of Agriculture, University of Peradeniya, Peradeniya, Sri Lanka; ^4^Division of Global Epidemiology, Research Center for Zoonosis Control, Hokkaido University, Sapporo, Japan; ^5^Global Station for Zoonosis Control, Global Institution for Collaborative Research and Education, Hokkaido University, Sapporo, Japan

**Keywords:** recessive resistance, clustered regularly interspaced short palindromic repeats/Cas9, tomato, potato virus Y, cucumber mosaic virus, eIF4E

## Abstract

Eukaryotic translation initiation factors, including eIF4E, are susceptibility factors for viral infection in host plants. Mutation and double-stranded RNA-mediated silencing of tomato *eIF4E* genes can confer resistance to viruses, particularly members of the *Potyvirus* genus. Here, we artificially mutated the *eIF4E1* gene on chromosome 3 of a commercial cultivar of tomato (*Solanum lycopersicum* L.) by using CRISPR/Cas9. We obtained three alleles, comprising two deletions of three and nine nucleotides (3DEL and 9DEL) and a single nucleotide insertion (1INS), near regions that encode amino acid residues important for binding to the mRNA 5' cap structure and to eIF4G. Plants homozygous for these alleles were termed 3DEL, 9DEL, and 1INS plants, respectively. In accordance with previous studies, inoculation tests with potato virus Y (PVY; type member of the genus *Potyvirus*) yielded a significant reduction in susceptibility to the N strain (PVY^N^), but not to the ordinary strain (PVY^O^), in 1INS plants. 9DEL among three artificial alleles had a deleterious effect on infection by cucumber mosaic virus (CMV, type member of the genus *Cucumovirus*). When CMV was mechanically inoculated into tomato plants and viral coat accumulation was measured in the non-inoculated upper leaves, the level of viral coat protein was significantly lower in the 9DEL plants than in the parental cultivar. Tissue blotting of microperforated inoculated leaves of the 9DEL plants revealed significantly fewer infection foci compared with those of the parental cultivar, suggesting that 9DEL negatively affects the initial steps of infection with CMV in a mechanically inoculated leaf. In laboratory tests, viral aphid transmission from an infected susceptible plant to 9DEL plants was reduced compared with the parental control. Although many pathogen resistance genes have been discovered in tomato and its wild relatives, no CMV resistance genes have been used in practice. RNA silencing of *eIF4E* expression has previously been reported to not affect susceptibility to CMV in tomato. Our findings suggest that artificial gene editing can introduce additional resistance to that achieved with mutagenesis breeding, and that edited *eIF4E* alleles confer an alternative way to manage CMV in tomato fields.

## Introduction

Tomato, which originated in Latin America, belongs to the Solanaceae family. This family consists of approximately 100 genera and 2,500 species, including other agronomically important plants, such as potato (*Solanum tuberosum* L.), pepper (*Capsicum*), and tobacco (*Nicotiana tabacum* L.; Olmstead et al., 2008). Tomato has a rich nutrient profile. As one of the major globally grown crops, annual global production exceeds 160 million tons, accounting for more than 10% of global vegetable production.^1^Tomato is susceptible to more than 200 diseases, which are caused by a great variety of pathogens including viruses, bacteria, fungi, and nematodes (Lukyanenko, 1991). Because the use of agricultural chemicals is often ineffective, especially for plant viruses, and expensive for growers, the breeding of resistant crops is important for sustainable crop production.

Cucumber mosaic virus (CMV) has been ranked among the top five scientifically and economically important plant viruses (Scholthof et al., 2011). In the field, this virus is readily transmitted in a stylet-borne, nonpersistent manner by more than 80 species of aphids; transmission may occasionally be seed-borne. CMV can negatively affect plant growth and fruit yield, with associated mosaic or lethal necrotic symptoms. Although the losses are difficult to quantify because the viral incidence changes annually in different locations, tomato yield losses caused by CMV infection have been reported to reach 25–50% of the total yield in China (Tien and Wu, 1991) and 80% of the total yield in Italy and Spain (Gallitelli et al., 1991; Jordá et al., 1992).

Although many genes that convey resistance to pathogens have been discovered in tomato and its wild relatives (Foolad, 2007), the few genes that have been shown to promote resistance to CMV have not been practically used in tomato breeding (Stamova and Chetelat, 2000). Consequently, many trials of ways to improve resistance have been performed. These include investigations where CMV gene was introduced into the host by genetic engineering, and antiviral resistance was achieved by RNA silencing (Anderson et al., 1992; Palukaitis et al., 1992; Yie et al., 1992) and the practical development of an attenuated CMV strain containing satellite RNA (Sayama et al., 1993).

Studies of resistance in plant breeding over the past 2 decades have identified not only dominant but also recessive resistance genes (Gallois et al., 2018). Plant viruses are obligate parasites. Their replications depend on host plant because viral genomes are smaller than those of other plant pathogens except viroids and contain only a dozen or fewer genes (Sanfacon, 2017). A plant that harbors a loss-of-function mutation in an opportune host factor but can complete the viral replication becomes resistant to the virus through inheritance of a recessive trait (Hashimoto et al., 2016; Bastet et al., 2017). Examples of recessive resistance have been demonstrated for mutant alleles of genes of the eukaryotic translation initiation factor (eIF) family. Loss-of-function studies on the *eIF4E* gene isoform in *Arabidopsis thaliana* have shown that resistance is conferred against members of the genus *Potyvirus* (Lellis et al., 2002; Sato et al., 2005). Several similar cases of eIF4E-associated resistance against other viruses in various host species from different families have been reported, including barley yellow mosaic virus in rice (*Oryza sativa*; Albar et al., 2006), CMV and turnip crinkle virus in *A. thaliana* (Yoshii et al., 2004), and melon necrotic spot virus in melon (*Cucumis melo*) (Nieto et al., 2006).

Natural antiviral alleles of *eIF4E1* have been identified as *pot1* and *pot1*^2^ on chromosome 3 in tomato relatives (Ruffel et al., 2005; Lebaron et al., 2016). A knockout mutant of *eIF4E2* on chromosome 2 confers resistance to pepper veinal mottle virus (Piron et al., 2010; Moury et al., 2020). Interestingly, *pot1* shows a wider spectrum of resistance to members of the *Potyvirus* genus than does a knockout mutant of eIF4E1; in addition, it shows a similar spectrum to that of eIF4E1 and eIF4E2 double-knockout, without the growth defect seen in the double-knockout plant (Gauffier et al., 2016). RNA silencing of eIF4E expression also leads to a wider spectrum of resistance to members of the genus *Potyvirus*, but not to other viruses, including CMV (Mazier et al., 2011). Mutagenesis breeding has been carried out widely by using physical and chemical mutagens to randomly mutagenize a large population and then select mutants that show the target traits (Sikora et al., 2011; Oladosu et al., 2016). Recently genome editing techniques based on the bacterial immune system (Clustered Regularly Interspaced Short Palindromic Repeats; CRISPR/Cas9 system; Jinek et al., 2012) have become very useful tools for efficiently obtaining loss-of-function mutants (Bortesi and Fischer, 2015; Jaganathan et al., 2018). In the breeding of crops for resistance, CRISPR/Cas9-mediated mutagenesis has been shown to be a practical method for obtaining mutations in eIFs that confer viral resistance in cucumber (*Cucumis sativus L.*) and rice (*Oryza sativa*; Chandrasekaran et al., 2016; Macovei et al., 2018). Here, by applying CRISPR/Cas9 to edit the *eIF4E1* gene on chromosome 3 in a commercial tomato cultivar, we obtained additional alleles of *eIF4E1*. Tomato plants carrying these alleles showed differential resistance not only to potato virus Y (PVY) but also to CMV.

## Materials and Methods

### Binary Plasmid Construction

**Figure 1:**
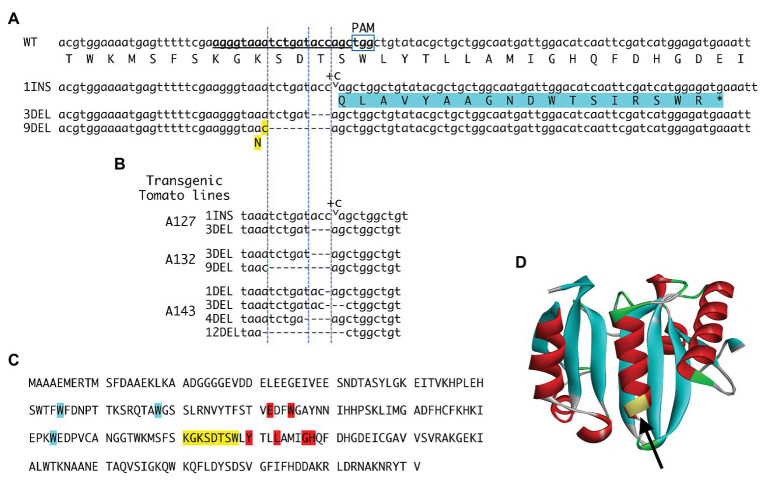
eIF4E1 alleles artificially created *via* CRISPR/Cas9. **(A)** Wild-type (WT) sequence and sequences of the new alleles. Bold underlined letters indicate the guide RNA (gRNA) target sequence. The protospacer adjacent motif (PAM) is marked. Three alleles were obtained: a one-cytosine insertion (1INS), a three-nucleotide deletion (3DEL), and a nine-nucleotide deletion (9DEL). The 1INS (marked by the arrowhead and + C) induces a − 1 frameshift immediately after the 144th codon, assigning threonine in the translation of *eIF4E1*; this results in premature truncation of the frameshift-mediated extending peptide (highlighted in blue). Protein translated from the 3DEL allele has a deletion of threonine encoded by the 144th codon, and the protein translated from the 9DEL allele has a single amino acid substitution (lysine to asparagine at the X codon; highlighted in yellow) and a deletion of three amino acid residues, serine, aspartic acid and threonine. **(B)** Mutations in the eIF4E1 gene in three transgenic tomato plants harboring the CRISPR/Cas9 construct were investigated by amplicon sequencing. In addition to the above three alleles **(A)**, alleles with 1-, 4-, and 12-nucleotide deletions (1DEL, 4DEL, and 12DEL) were detected. **(C)** The amino acid sequence of eIF4E1. The amino acids corresponding to the gRNA nucleotide sequence are highlighted in yellow and are in close proximity to the amino acids that are critical for binding to the mRNA 5' cap structure (highlighted in blue) and the eIF4G protein (highlighted in red; German-Retana et al., 2008; Miras et al., 2017). **(D)** Three-dimensional structure of the tomato eIF4E1 protein. The region indicated with an arrowhead corresponds to that targeted by the gRNA sequence.

This study used two vectors, pUC19-AtU6oligo and pZK_gYSA_FFCas9 (Mikami et al., 2015; Kanazashi et al., 2018), to create the binary vector pZK_AtU6gRNA_FFCas9_NPTII, following the method described by Osmani et al. (2019). Guide RNA (gRNA) recognition sites in tomato were found using CRISPRdirect.^2^ The sense and antisense oligonucleotide sequences (5'-attgagggtaaatctgataccagc-3' and 5'-aaacgctggtatcagatttaccct-3', respectively), including the nucleotide sequence of a target site in the *eIF4E1* gene on chromosome 3 (underlined in Figure 1A), were synthesized and annealed to a pair of oligonucleotides for the gRNA to *eIF4E1*. The annealed oligonucleotides were cloned into the BbsI sites of pUC19_AtU6oligo. Then, the cloned plasmid was digested with restriction enzyme I-SceI, and the gRNA expression cassette was re-cloned into pZD_AtU6gRNA _FFCas9_NPTII. *Agrobacterium tumefaciens* LBA4404 (Takara Bio, Shiga, Japan) was then transformed with the binary vector.

### Plant Transformation and Transgenic Plant Propagation

Kikkoman Corporation’s inbred line S8, a big-fruited tomato, was used for artificial editing by CRISPR/Cas9. Plants were transformed as described by Sun et al. (2006). In brief, diced leaves were inoculated with *A. tumefaciens* LBA4404 transformed with the constructed binary plasmid vector. Callus was generated, and shoots were formed. The shoots were transferred to and grown on a selective regeneration medium containing 1.5 mg/L zeatin, 50 mg/L kanamycin, and 200 mg/L carbenicillin in a growth chamber (16 h light/8 h dark). Then the largest shoots were transferred to a rooting medium containing 50 mg/L kanamycin, 100 mg/L carbenicillin, and 0.1 mg/L 1-naphthylacetic acid. Lines with roots were transferred for culture in soil pots under white fluorescent light (14 h light/10 h dark). T1, T2, and T3 generations of the transgenic lines were produced through self-pollination of each parent generation.

### Genotyping of Transgenes and Mutations

Tomato genomic DNA was extracted from transgenic and non-transgenic regenerated plants by using a MonoFas Plant DNA Extraction Kit (GL Sciences, Tokyo, Japan). The presence of the transgene including the gRNA region was checked by polymerase chain reaction (PCR) analysis using a pair of primers (5'-tgggaatctgaaagaagagaagca-3' and 5'‐aaacgctggtatcagatttaccct-3') and KOD-FX enzyme solution (Toyobo, Osaka, Japan). PCR conditions were incubation at 95°C for 2 min, followed by 30 cycles of 98°C for 10 s, 60°C for 25 s, and 68°C for 15 s, with a final step of 68°C for 3 min. Subsequently, the transgenic lines were genotyped for mutations at the *eIF4E* target sites by using a cleaved amplified polymorphic sequences (CAPS) assay as follows. PCRs were performed under the above conditions, but using a different pair of primers (5'‐ atccatcacccaagcaagttaatt-3' and 5'-gtccacaaagctattttttctccc-3'), and the PCR products were digested with the restriction enzyme PvuII. The digested products were separated using 1.8% agarose gel electrophoresis. The indel mutations in T1 and T2 progeny seedlings were confirmed by sequencing the target region. PCR fragments from the CAPS assay were cloned into the pTA2 vector (Toyobo), and nucleotide sequences of several clones were determined.

### Three-Dimensional Structure Modeling

The three-dimensional structure of tomato eIF4E1 was constructed by homology modeling based on the crystal structure of *Pisum sativum* eIF4E (Protein Data Bank ID: 2WMC) by using Discovery Studio 2017 software (Biovia).

### Mechanical Inoculation and Detection of the Viruses

Cucumber mosaic virus strain yellow (CMV-Y) and PVY strains N and O (PVY^N^ and PVY^O^) (Kwon et al., 2020) were initially propagated on *Nicotiana benthamiana*, and infected upper leaves were used as inoculum for mechanical inoculation. Tomato seedlings at the second or third true leaf stage were mechanically inoculated with crude inoculum, which was viral-infected leaf tissue ground in 0.1 M phosphate buffer (pH 7.0). Following the time-course observations of symptom expression, viral coat protein (CP) accumulation in non-inoculated upper leaves at 25 or 35 days post inoculation (dpi) was investigated with a double antibody sandwich-enzyme linked immunosorbent assay (ELISA) using anti-CP polyclonal antibody (Japan Plant Protection Association). Reverse transcription PCR (RT-PCR) analysis to detect CMV-Y genomic RNA was performed with a pair of primers, 5'-gtacagagttcagggttgagcg-3' and 5'-agcaatactgccaactcagctcc-3', as described in our previous study (Kwon et al., 2020).

### Blotting of Microperforated Leaves Inoculated With CMV-Y

Tissue blotting was performed as described previously (Kaneko et al., 2004; Murakami et al., 2016); anti-CMV CP polyclonal antibody was used. Sizes of infection foci and infected area were measured using ImageJ (Wayne Rasband, National Institutes of Health). Focus size was measured for foci whose area ranged from 0.1 to 30 mm^2^. Focus number was calculated for foci whose size ranged from 0.001 to 30 mm^2^.

### Aphid Inoculation With CMV-O and Evaluation of Transmission Efficiency

*Myzus persicae* was grown on healthy *Brassica rapa*, and the ordinary strain of CMV (CMV-O) was used for the aphid transmission assay, since CMV-Y is known to have defects in transmission *via* aphids. To assess the infection frequency of *M. persicae*, apterous aphids that had been starved for 3 h were allowed a 2–5 min acquisition access period on CMV-O-infected *N. tabacum* and tomato plants at 10–30 dpi. Ten aphids were collected and transferred to each genome-edited and wild-type (WT) S8 tomato plant with the use of a fine dry paintbrush. After 1 day on the tomato plant, the aphids were then killed by pesticide. CMV infection was confirmed by the PCR analysis described below and by the assessment of visual symptoms, yellowing, mosaic, and ringspot of leaves. Viral RNA was extracted by TRIzol reagent (Thermo Fisher Scientific, Waltham, MA, United States), and first-strand cDNA was synthesized using AMV reverse transcriptase (Promega, Madison, WI, United States). PCR was performed using cDNA corresponding to 50 μg of RNA extract, 0.5 μM each primer (5'-gtacagagttcagggttgagcg-3', 5'-agcaatactgccaactcagctcc-3'), and Ex Taq polymerase (Takara Bio). The PCR was carried out under the conditions of 2 min at 95°C, followed by 35 cycles of 20 s at 95°C, 30 s at 55°C, and 30 s at 72°C, with a final step of 5 min at 72°C. The PCR products were fractionated using 1.8% agarose gel electrophoresis.

## Results

### Development of Artificial Mutated eIF4E Alleles in a Commercial Tomato Line

Tomato has two *eIF4E* genes, *eIF4E1* and *eIF4E2*, on chromosomes 3 and 2, respectively; and an isoform *eIF(iso)4E* on chromosome 9. Here we transformed a big-fruited tomato line S8, with *A. tumefaciens* harboring the binary vector for CRISPR-mediated editing of *eIF4E1*. The region targeted by the gRNA (underlined, Figure 1A) corresponds to the yellow-highlighted amino acid sequence in Figure 1C and to the part of the three-dimensional structure indicated by the arrow in Figure 1D. The targeted region is near the amino acid residues critical for binding to the mRNA cap (7-methylguanosine) and eIF4G (Figure 1C, highlighted in blue and red, respectively) (German-Retana et al., 2008; Miras et al., 2017). Thus, mutations in the targeted region may affect binding to these entities. The CAPS assay for the targeted sites identified 20 transformants with artificially edited *eIF4E1* alleles. T1 progenies were derived from self-pollination of three out of 20 T0 regenerated plants, and generated mutations in the targeted *eIF4E* loci in their genomes were investigated (Figure 1B). DNA fragments including the targeted sites were amplified by PCR and cloned. As a result of nucleotide sequencing of these clones, six artificial *eIF4E1* alleles were detected in T1 plants (Figure 1B), including insertion of one nucleotide (1INS) and deletion of three and nine nucleotides (3DEL and 9DEL, respectively). The allele 1INS causes a − 1 frameshift immediately after the 144th codon assigning threonine in the translation of eIF4E1 and results in premature truncation of the frameshift-mediated extending peptide. The alleles 3DEL and 9DEL encode the proteins that have a deletion of threonine at the 144th codon and serine, aspartic acid, and threonine, following an amino acid substitution from lysine to asparagine (N in Figure 1A, highlighted in yellow). All cloned PCR fragments (a total of 16 clones) for which nucleotide sequences were determined corresponded to one of the above-mentioned artificial alleles but not the WT allele, suggesting that *eIF4E1* was bi-allelically edited by CRISPR/Cas9 in the T0 generation. Furthermore, null-segregant progenies, without transgene, were obtained from the T1 or T2 generation. The mutant plants showed almost the same phenotype and traits as WT tomato in enclosed greenhouse cultivation (data not shown).

### Distinct Susceptibility to PVY^N^ and PVY^O^ in Homozygotes of the Artificial *eIF4E1* Alleles

**Figure 2:**
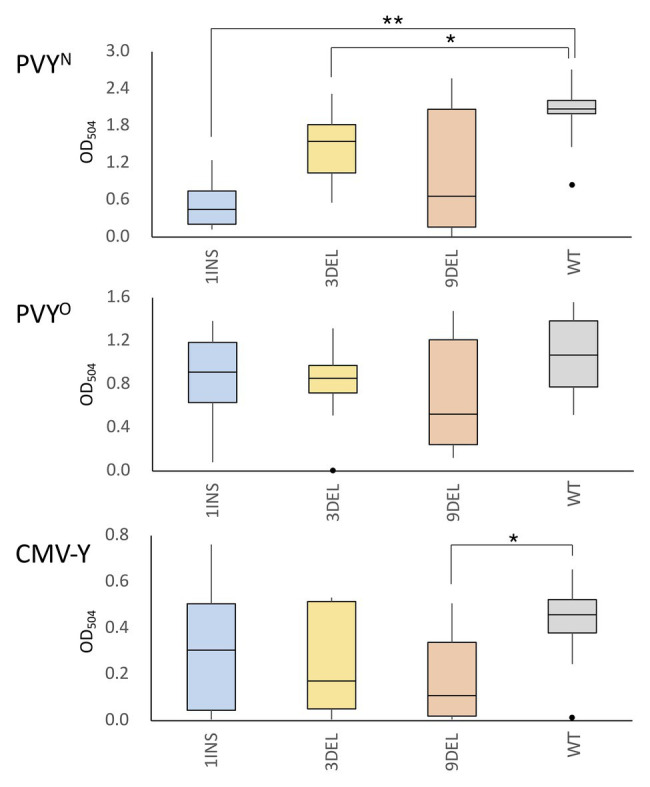
Accumulation of potato virus Y (PVY) and cucumber mosaic virus (CMV) coat protein (CP) in the non-inoculated upper leaves of tomato plants carrying the artificial *eIF4E1* alleles. PVY^N^ was mechanically inoculated into plants homozygous for the *eIF4E1* alleles (1INS, *n* = 8; 3DEL, *n* = 12; 9DEL, *n* = 7) or the parental line S8 (WT; *n* = 14). Similarly, plants were inoculated with PVY^O^ or CMV-Y (PVY^O^ experiment: 1INS, *n* = 10; 3DEL, *n* = 10; 9DEL, *n* = 7; and WT, *n* = 8; CMV-Y experiment: each allele, *n* = 10, WT, *n* = 10). Accumulation of PVY and CMV CP was investigated using enzyme linked immunosorbent assay (ELISA) with anti-PVY or anti-CMV CP polyclonal antibodies at 25 and 35 days post inoculation (dpi), respectively. Data are presented as boxplots. ^*^p < 0.05; ^**^p < 0.01 (Wilcoxon’s signed-rank test).

Since the eIF4E protein is a host susceptibility factor that plays a pivotal role in potyviral infection, its recessive resistance alleles are considered to lack the function that supports infection. Therefore, plants become resistant to the virus only when the natural resistance alleles (including the *pot1* allele of tomato *eIF4E1*) are homozygous (Hashimoto et al., 2016; Bastet et al., 2017). We inoculated tomato plants that were homozygous for one of the above three artificially edited alleles with PVY^N^ or PVY^O^. These strains cause systemic veinal necrosis and mottle, respectively, in tobacco, and both systemically and asymptomatically infect WT tomato plants. The results of ELISA with anti-PVY CP antibody showed lower accumulation of PVY^N^ CP in the non-inoculated upper leaves in plants homozygous for the artificial *eIF4E1* alleles than in WT plants. This difference was statistically significant for the 1INS and 3DEL alleles, but not the 9DEL allele, and was particularly marked for the 1INS allele, which is considered to confer loss-of-function through frame-shifting and the introduction of a premature stop codon during translation (Figure 2). In contrast, PVY^O^ CP accumulated comparably in the artificial allele homozygotes and parental WT cultivar, suggesting that functional eIF4E1 protein is required for infection with PVY^N^ but not PVY^O^.

### Reduced Susceptibility to CMV in Tomato Carrying the Artificial *eIF4E1* Alleles

The loss-of-function mutant of *Arabidopsis* eIF4E, *cum1*, is less susceptible to CMV than WT *Arabidopsis* (Yoshii et al., 2004). Therefore, we next examined whether CMV infection was controlled in tomato plants with the artificial *eIF4E1* alleles. For each of the three edited alleles, a dozen T2 plants homozygous for the allele were mechanically inoculated with CMV-Y. Most inoculated plants showed symptoms at 30 dpi, with or without the artificial eIF4E1 allele. Investigation of CMV CP accumulation in the non-inoculated upper leaves by ELISA using anti-CMV CP antibody showed that the levels of CP accumulation in plants carrying 9DEL were significantly lower than those in the parental WT plants; no significant difference was found for the other alleles. This suggests that tomato *eIF4E1* is involved in CMV infection and that 9DEL negatively affects CMV infection in tomato (Figure 2).

### Resistance of 9DEL Tomato Plants to Mechanically Inoculated CMV

**Figure 3:**
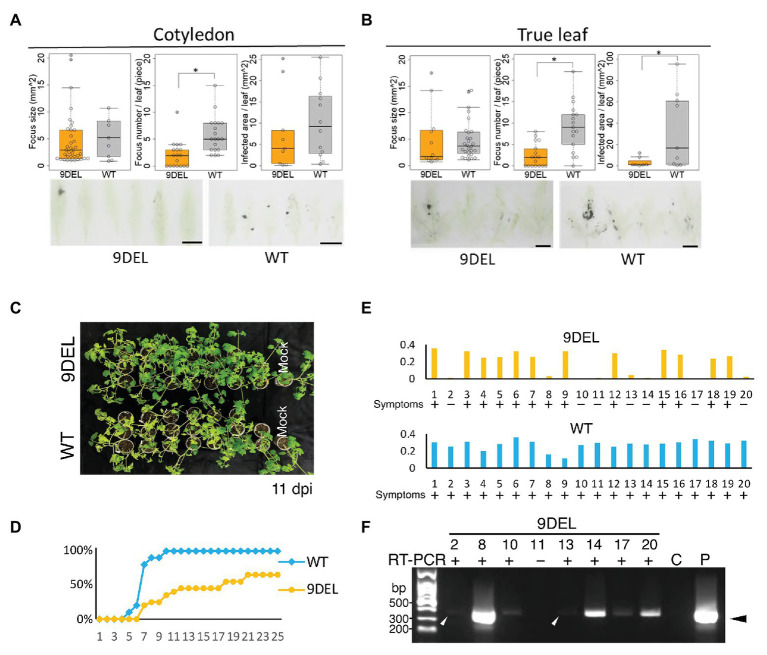
Detailed investigation of resistance to CMV-Y in 9DEL plants. Infection foci in inoculated cotyledons **(A)** and true leaves **(B)** were detected by microperforated leaf blotting with anti-CMV CP antibody. **(A)** The focus size [9DEL, *n* = 9; parental cultivar (WT), *n* = 37], focus number per leaf (9DEL, *n* = 18; WT, *n* = 19), and infected area per leaf (9DEL, *n* = 9; WT, *n* = 12xx) were compared between cotyledons of 9DEL plants and WT. **(B)** The focus size (9DEL, *n* = 12; WT, *n* = 28), focus number per leaf (9DEL, *n* = 17; WT, *n* = 17), and infected area per leaf (9DEL, *n* = 7; WT, *n* = 9) were compared between true leaves of 9DELplants and WT. Data are presented as boxplots and individual data points. ^*^p < 0.05 (Student’s *t*-test). **(C)** Images of inoculated and mock-inoculated plants at 11 dpi are shown. Symptoms are yellowing of upper leaves. **(D)** Time-course of percentage of inoculated plants displaying symptoms (*n* = 20). **(E)** Results of ELISA of CMV CP accumulation in the upper leaves at 34 dpi. The data were corrected by subtracting the level in a corresponding healthy plant from the raw data. Data for 20 individual plants are displayed for each genotype. **(F)** Reverse transcription-polymerase chain reaction (RT-PCR) analysis of CMV genomic RNA in the upper leaves of the eight symptomless 9DEL plants. Control samples were prepared from healthy (C) and infected (P) tomato leaves. Arrowheads indicate the position of the expected band by RT-PCR.

We then investigated which steps are affected by 9DEL during the infection cycle of CMV (Figure 3). By tissue blotting of microperforated inoculated leaves using anti-CMV CP antibody, we found that there was a significantly smaller infection area per inoculated true leaf in 9DEL plants than in WT plants (Figures 3A,B right graphs); this difference was mainly due to a significantly lower infection focus number per leaf in 9DEL plants because there was no significant difference in focus size (Figures 3A,B left and middle graphs). All inoculated parental cultivar plants showed symptoms, including severe yellowing in the upper leaves by 11 dpi, whereas the inoculated 9DEL plants showed no or few symptoms in the upper leaves at this time (Figure 3C), and one-third (7/20) of the 9DEL plants did not show any symptoms by 35 dpi (Figure 3D). By conducting ELISA to detect CMV CP, we demonstrated that the symptomless inoculated 9DEL plants contained an undetectable level of CMV CP (comparable or less than that in the control mock-infected plant) in the upper leaves at 35 dpi (Figure 3E). When the symptomless inoculated 9DEL plants were subjected to RT-PCR analysis, CMV genomic RNA was detected in six-sevenths of plants (Figure 3F), indicating their latent systemic infection with CMV. These results indicate that 9DEL allows systemic infection with CMV although it inhibits the establishment of CMV infection in the mechanically inoculated leaves.

### Resistance to Infection by Aphid Transmission of CMV in Tomato Carrying 9DEL

**Figure 4:**
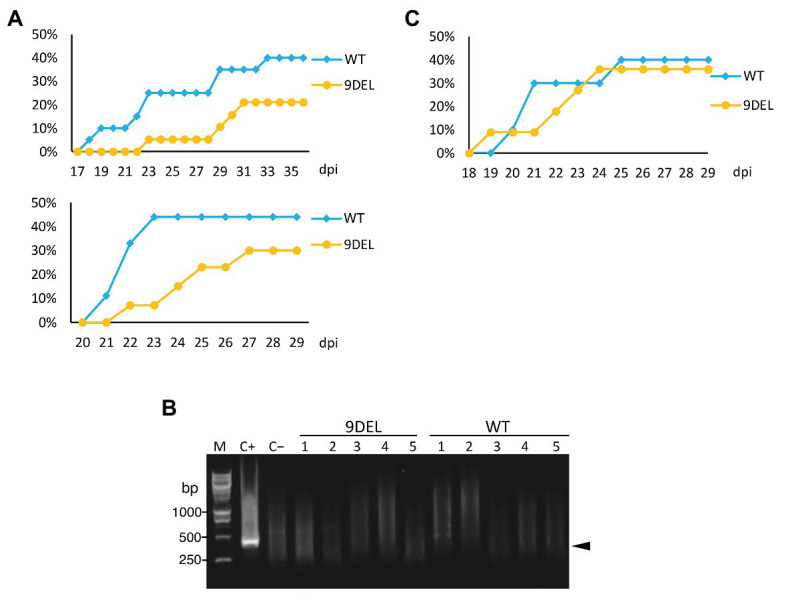
Aphid transmission of CMV in 9DEL plants compared with the parental line **(A)** CMV-O was transmitted from an infected tobacco plant to 9DEL plants (*n* = 19) or the parental line (WT). The percentage of plants with symptomatic leaves over time is shown for two independent experiments. **(B)** RT-PCR was conducted to detect CMV genomic RNA in five of each of the 9DEL and WT plants that did not show symptoms. C+, control plant showing symptoms; C−, healthy tomato plant. M, DNA size markers. **(C)** CMV-O was transmitted from an infected 9DEL or WT plant to healthy WT plants (*n* = 10). The percentage of plants with mosaics and abnormal leaves over time is shown.

To determine whether the 9DEL allele is useful for CMV management in practice, we tested the efficiency of aphid transmission of CMV from infected *N. tabacum* to 9DEL tomato plants (Figure 4). When we monitored the number of plants with mosaics and abnormal leaves over time, we obtained similar results in two separate experiments (Figure 4A): i.e., the aphid transmission of CMV was reduced to around half of the level observed in WT tomato (Figure 4A). In addition, RT-PCR results revealed that the non-symptomatic plants were not actually infected by CMV (Figure 4B). These results indicate that the 9DEL mutation resulted in immunity as opposed to tolerance (Figures 3C-F). We then compared aphid transmission from an infected 9DEL plant vs. infected WT plant to a WT plant. Infected 9DEL and WT plants that showed symptoms were used as the aphid source. Aphid transmission from the infected 9DEL and WT plants showed similar efficiency to each other (Figure 4C).

## Discussion

Disease-related yield loss is a critical issue for farmers. Breeding disease-resistant crops with maintained or improved yields and reduced pesticide dependence is important for sustainable agriculture. Although tomatoes have been bred for resistance against viruses, no such work has been undertaken for CMV because no genetic resource is available (see “Introduction”). Here, we used artificial gene editing of eIF4E to introduce additional resistance against PVY and CMV in a commercial tomato cultivar.

In most cases, natural mutants and genetically modified alleles of eIF4E in tomato and other crops confer resistance to members of the genus *Potyvirus*, such as PVY (see Introduction). In general, plants possess multiple genes for eIF4E and its isoforms, and *Potyvirus* members do not necessarily use a single eIF4E for their infection (Bastet et al., 2017); the particular eIF4E gene(s) required for infection with a virus differ between host plant species. We initially tested the effect of our artificially edited *eIF4E1* alleles in tomato on susceptibility to two strains of PVY, PVY^N^, and PVY^O^. The 1INS and 3DEL alleles significantly reduced the susceptibility to PVY^N^ but not to PVY^O^ (Figure 2). These results can be interpreted on the basis of previous studies in which eIF4E1 and eIF4E2 were individually or doubly knocked out or down in tomato (Mazier et al., 2011; Gauffier et al., 2016; Bastet et al., 2017; Moury et al., 2020). The previous studies indicated that PVY primarily uses eIF4E1, but most PVY strains can alternatively use eIF4E2 to promote infection when eIF4E1 is knocked out or down. Our results suggest that PVY^O^ is one of the major PVY strains that can alternatively use eIF4E2, whereas PVY^N^ is one that exclusively uses eIF4E1 to promote infection. Therefore, 1INS, a frameshift mutation, appears to make eIF4E1 non-functional for PVY infection. Viruses of the family *Potyviridae* have a viral protein genome-linked (VPg) covalently attached at the 5' end of their RNA genomes instead of the 7-methylguanosine cap structure that is found at the 5' end of some eukaryotic and viral mRNAs. VPg has affinity to host eIF4E and its isoform not only for translation of viral proteins but also for viral RNA stabilization and evading antiviral RNA silencing (Saha and Makinen, 2020); the degree of affinity is correlated with viral infectivity (Leonard et al., 2000). Therefore, significant reduction of susceptibility to PVY^N^ in tomato carrying 3DEL implies the possibility that the deletion mutation of 3DEL affects the affinity to PVY^N^ VPg. Docking models between eIF4E1s translated from the artificially edited alleles with the Cap structure (Supplementary Figure S1A) and VPg (Supplementary Figure S1B) suggest that deletions and substitution of both 3DEL and 9DEL disrupt eIF4E1 binding to both Cap and VPg. Our finding that the *eIF4E1* region deleted in 9DEL encodes amino acid residues important for binding to cap structures implies that the reduced susceptibility to CMV in tomato carrying 9DEL could be attributed to a defect eIF4E1 binding to the 5' caps of CMV RNAs. These results also suggest that we can design non-transgenic virus-resistant plants by using the genome-editing technology, on the basis of the *in silico* estimation of the interactions between host factors and viral proteins.

Previously, RNA silencing of both *eIF4E1* and *eIF4E2* conferred broad resistance against members of the genus *Potyvirus* but not against other viruses, such as tomato spotted wilt virus, alfalfa mosaic virus, tobacco mosaic virus, and CMV (Mazier et al., 2011). However, in the current study, editing of a single *eIF4E* gene, *eIF4E1*, to produce the 9DEL allele, negatively affected CMV multiplication and infection efficiency by aphid transmission. This apparent discrepancy may be attributed to a dominant effect of the mutant eIF4E1 protein expressed by this allele. The natural mutant allele of the *eIF4E1* gene, *pot1*, was identified in a wild relative of tomato (Ruffel et al., 2005). The *eIF4E1* knockout allele, which expresses a truncated protein lacking cap-binding activity, was obtained by ethyl methanesulfonate mutagenesis (Piron et al., 2010). Plants homozygous for both *pot1* and the knockout allele show resistance to members of the genus *Potyvirus*. Recent studies compared the resistance spectra among plants carrying *pot1*, the *eIF4E1* knockout allele, or both *eIF4E1* and *eIF4E2* double-knockout alleles (Gauffier et al., 2016; Bastet et al., 2017). The *pot1* homozygous and double-knockout plants of *eIF4E1* and *eIF4E2* showed resistance to a higher number of members of the genus *Potyvirus* compared with *eIF4E1* knockout homozygous plants, even though the *pot1* product has four amino acid substitutions and seems to retain the essential functions of eIF4E. Their analyses suggest that the *pot1* product is not only functionless for *Potyvirus* infection but also dominant-negatively suppresses eIF4E2 expression and thus confers a similar resistance spectrum as the double knockout. The 9DEL allele in this study may have a similar dominant-negative effect on the genes required for CMV multiplication and transmission. The 9DEL allele reduced CMV susceptibility more than the 1INS allele, which we consider to be a knockout mutant because the deduced truncated translation product lacks the critical domain to bind to eIF4G (Figure 1B).

Almost all mechanically inoculated tomato plants carrying the artificially generated *eIF4E1* alleles were infected with CMV, but 9DEL plants accumulated a lower amount of CMV CP in their upper leaves than that in WT plants (Figure 2). In our detailed investigation of the differences in resistance to CMV between 9DEL and WT plants (Figure 3), one-third of inoculated 9DEL plants did not develop any symptoms when the accumulation of CMV CP in the upper leaves was low (Figure 3E). Although most of the symptomless plants still allowed systemic infection with CMV (Figure 3F), there were significantly fewer foci in the inoculated leaves of the 9DEL plants that the WT plants (Figures 3A,B), suggesting that 9DEL negatively affects the initial establishment of CMV infection. Incomplete resistance to CMV was previously reported in *A. thaliana*. When the eIF4E knockout mutant *cum1* of *A. thaliana* was inoculated with CMV, the mutant inhibited the expression of CMV movement protein but still accumulated a similar amount of the other viral proteins and RNAs to those of WT (Yoshii et al., 2004). Here, the expression of the movement protein and CMV movement may have been affected in the tomato plants carrying the edited alleles, although recent studies indicate that eIF4E is involved in multiple steps of various types of viral infection or propagation (Montero et al., 2015). CMV was still detected by RT-PCR in most sample RNAs from mechanically inoculated plants without symptoms (Figure 3F), but CMV RNAs were not detected in the 9DEL plants that did not show any symptoms in the aphid transmission trial (Figure 4B). On the basis of these results, we assume that the efficacy of the resistance provided by the 9DEL allele depends on the amount of invading CMV virions in the initially inoculated cells, and that when the invading CMV amount is small, the 9DEL allele completely inhibits systemic infection of CMV.

This study is the first report to demonstrate the possibility of introducing resistance to CMV by genome editing. Unlike conventional random mutagenesis, recently developed genome editing techniques are capable of efficiently mutagenizing a specific target gene. Direct genome editing of commercial crop cultivars reduces the time required for altering their traits. Although recessive resistance or natural mutants of host-susceptibility factors such as eIF4E and eIF4G are less utilized for breeding than dominant resistance mutants, some susceptibility factors including eIF4E are thought to be required for most virus infections, implying the possibility that one recessive resistance gene confers resistance to multiple different viruses at once. Considering the emergence of resistance-breaking races or strains of pathogens (Tsuda et al., 1998; Enya et al., 2009), we need to continuously explore and develop resistance genes by taking advantage of recent genetic engineering techniques, including genome editing approaches. The stacking of incomplete-resistance genes might result in sustainable resistance. Even the 9DEL plants were not able to completely inhibit CMV transmission by aphids in the laboratory trial in this study (Figure 4). Considering that exposure to CMV in natural fields is generally lower than in a laboratory trial (Hord et al., 2001; Kayode et al., 2014; Jalender et al., 2017), the 9DEL allele is likely to be of practical use for breeding cultivars with resistance to CMV.

## Data Availability Statement

The original contributions presented in the study are included in the article/Supplementary Material, further inquiries can be directed to the corresponding author/s.

## Author Contributions

HA, CM, and KN conceived and designed the experiments. HA, KN, and TY created CRISPR-edited tomato plants. HA, WJ, YT, JK, HK, and KN collected the samples and conducted analyses of the inoculated plants. HK, MI, and CM performed 3D and docking-modeling. HA, KI, CM, and KN discussed the results and drafted and revised the manuscript. All authors contributed to the article and approved the submitted version.

### Conflict of Interest

HA and KI were employed by the company Kikkoman Corporation.

The remaining authors declare that the research was conducted in the absence of any commercial or financial relationships that could be construed as a potential conflict of interest.

## Footnotes

^1^http://www.fao.org/home/en/

^2^https://crispr.dbcls.jp/
