# Supplementary material for: Artificially Edited Alleles of the Eukaryotic Translation Initiation Factor 4E1 Gene Differentially Reduce Susceptibility to Cucumber Mosaic Virus and Potato Virus Y in Tomato
Source: Front Microbiol. 2020 Dec 10;11:564310. doi: 10.3389/fmicb.2020.564310 (PMC7758215; doi:10.3389/fmicb.2020.564310)
Supplement: Supplementary file 1 [file Presentation_1.pptx]

## Slide 1
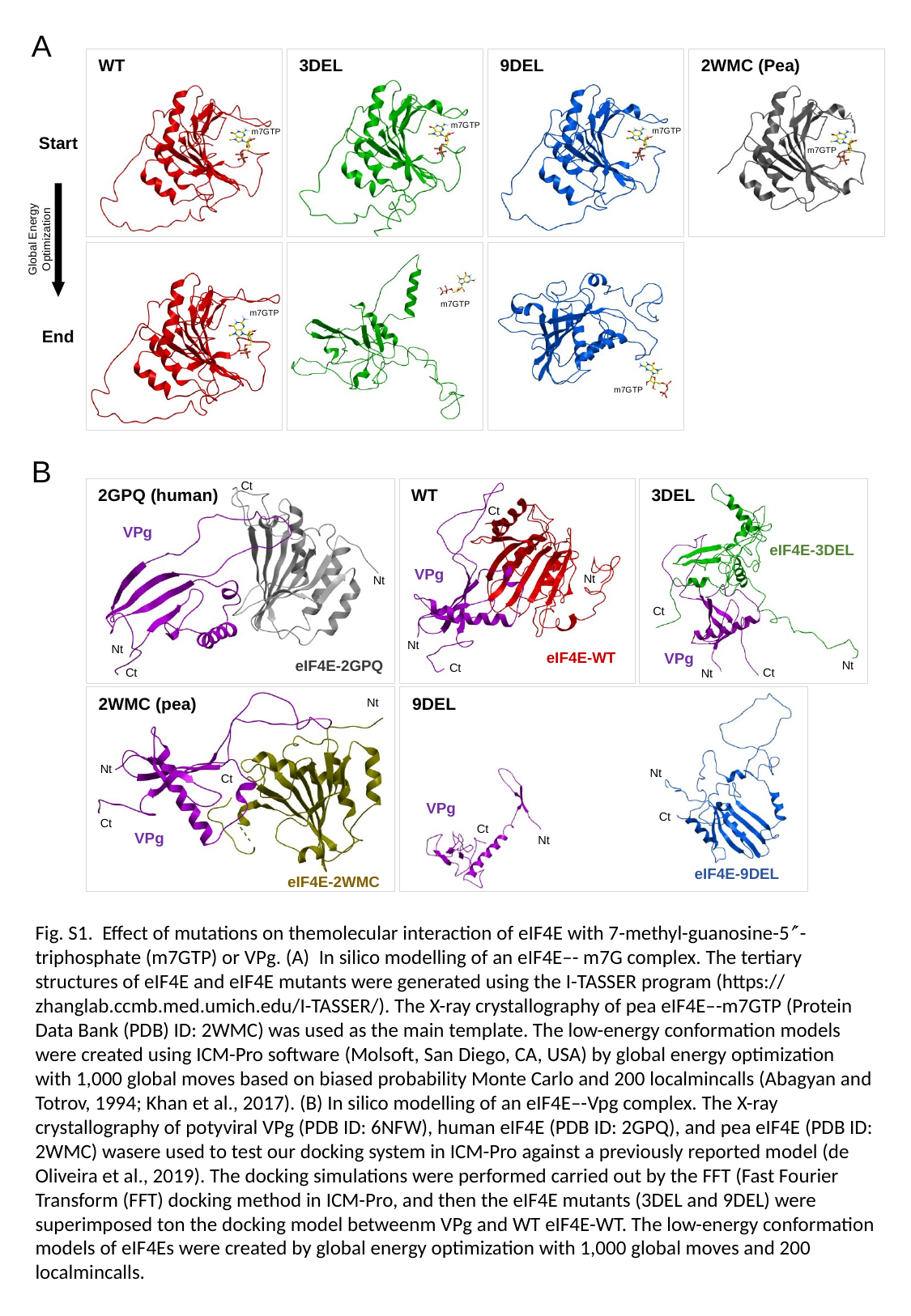

A
WT
3DEL
9DEL
2WMC (Pea)
m7GTP
m7GTP
m7GTP
Start
m7GTP
Global Energy
Optimization
m7GTP
m7GTP
End
m7GTP
B
Ct
2GPQ (human)
3DEL
WT
Ct
VPg
eIF4E-3DEL
VPg
Nt
Nt
Ct
Nt
Nt
eIF4E-WT
VPg
eIF4E-2GPQ
Nt
Ct
Ct
Ct
Nt
2WMC (pea)
9DEL
Nt
VPg
Ct
Ct
Nt
eIF4E-9DEL
Nt
Nt
Ct
Ct
VPg
eIF4E-2WMC
Fig. S1. Effect of mutations on themolecular interaction of eIF4E with 7-methyl-guanosine-5′´-triphosphate (m7GTP) or VPg. (A) In silico modelling of an eIF4E–- m7G complex. The tertiary structures of eIF4E and eIF4E mutants were generated using the I-TASSER program (https://zhanglab.ccmb.med.umich.edu/I-TASSER/). The X-ray crystallography of pea eIF4E–-m7GTP (Protein Data Bank (PDB) ID: 2WMC) was used as the main template. The low-energy conformation models were created using ICM-Pro software (Molsoft, San Diego, CA, USA) by global energy optimization with 1,000 global moves based on biased probability Monte Carlo and 200 localmincalls (Abagyan and Totrov, 1994; Khan et al., 2017). (B) In silico modelling of an eIF4E–-Vpg complex. The X-ray crystallography of potyviral VPg (PDB ID: 6NFW), human eIF4E (PDB ID: 2GPQ), and pea eIF4E (PDB ID: 2WMC) wasere used to test our docking system in ICM-Pro against a previously reported model (de Oliveira et al., 2019). The docking simulations were performed carried out by the FFT (Fast Fourier Transform (FFT) docking method in ICM-Pro, and then the eIF4E mutants (3DEL and 9DEL) were superimposed ton the docking model betweenm VPg and WT eIF4E-WT. The low-energy conformation models of eIF4Es were created by global energy optimization with 1,000 global moves and 200 localmincalls.

## Slide 2
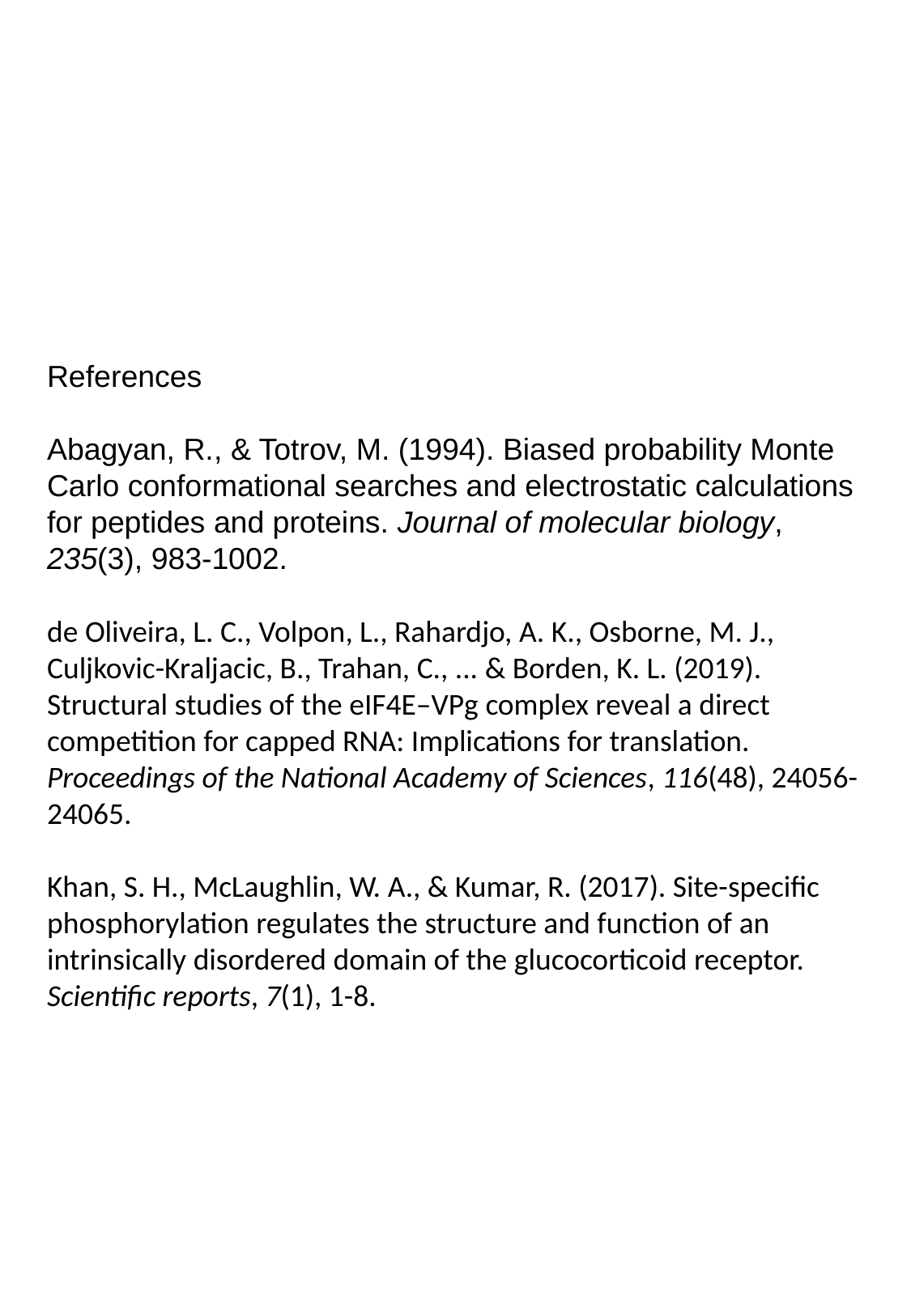

References
Abagyan, R., & Totrov, M. (1994). Biased probability Monte Carlo conformational searches and electrostatic calculations for peptides and proteins. Journal of molecular biology, 235(3), 983-1002.
de Oliveira, L. C., Volpon, L., Rahardjo, A. K., Osborne, M. J., Culjkovic-Kraljacic, B., Trahan, C., ... & Borden, K. L. (2019). Structural studies of the eIF4E–VPg complex reveal a direct competition for capped RNA: Implications for translation. Proceedings of the National Academy of Sciences, 116(48), 24056-24065.
Khan, S. H., McLaughlin, W. A., & Kumar, R. (2017). Site-specific phosphorylation regulates the structure and function of an intrinsically disordered domain of the glucocorticoid receptor. Scientific reports, 7(1), 1-8.
